# 奥雷巴替尼致伴血栓性微血管病的大量蛋白尿1例

**DOI:** 10.3760/cma.j.cn121090-20241120-00462

**Published:** 2025-06

**Authors:** 巧艳 张, 聪 刘, 英民 梁, 国辉 李

**Affiliations:** 西安国际医学中心医院，西安 710100 Xi'an International Medical Center Hospital, Xi'an 710100, China

患者，女，37岁。2018年12月于当地医院产检时查血常规示WBC 21.39×10^9^/L，后多次复查WBC波动于（25.29～33.32）×10^9^/L，未进一步诊治。2019年4月17日行骨髓细胞形态学及活检，结果示慢性髓性白血病（CML），染色体核型：46,XX,t（9；22）（q34；q11）,t（11；20）（q21；q13,3）；BCR::ABL融合基因（P210）阳性；诊断为CML（慢性期Sokal评分：低危），给予伊马替尼400 mg/d治疗。3个月后评估疗效为治疗失败，更换为达沙替尼100 mg/d治疗，1个月后患者出现双下肢水肿、腹痛、腹泻伴双眼视物模糊，考虑药物不耐受，更换为尼洛替尼300 mg，每日2次。

2019年10月行ABL1激酶区突变检测，检出T315I突变，遂参加临床试验，给予奥雷巴替尼40 mg隔日1次口服，3个月后达完全细胞遗传学反应，6个月疗效评估达主要分子学反应，持续口服奥雷巴替尼40 mg隔日1次治疗。

患者于2022年4月开始反复出现双下肢水肿，伴颜面水肿，晨轻暮重，休息后无明显缓解，无腰痛、尿频、尿急等不适。2022年9月就诊于我院，尿常规示蛋白（+++），尿比重1.016；24 h尿蛋白定量4 159.5 mg；肝肾功能检测示白蛋白32.7 g/L；肌酐59 µmol/L，肌酐清除率112.84 µmol/L；总胆固醇5.51 mmol/L（正常参考值<5.2 mmol/L），甘油三酯1.98 mmol/L（正常参考值<1.7 mmol/L），高密度脂蛋白1.44 mmol/L（正常参考值>1.04 mmol/L），低密度脂蛋白3.72 mmol/L（正常参考值<3.2 mmol/L）。血常规：WBC 3.2×10^9^/L，RBC 3.5×10^12^/L，HGB 108 g/L，PLT 69×10^9^/L。骨髓细胞染色体核型：46,XX[20]，BCR::ABL融合基因为0（ABL1拷贝数132 900）。双侧肾脏、输尿管及膀胱超声未见异常。完善肾穿刺活检病理提示：可见20个肾小球，其中4个肾小球节段性硬化，1个肾小球阶段性硬化，其余肾小球系膜细胞和基质轻度增生，基底膜弥漫增厚及双轨形成，少数肾小球毛细血管襻内有微血栓形成；肾间质轻度水肿，少量淋巴、单核细胞浸润及散在浆细胞浸润伴轻度纤维化（图1）。形态考虑急性肾小管间质损伤，符合血栓性微血管病。电镜示：基底膜节段性增厚，内皮下间隙明显增宽伴双轨征，其内可见絮状物，足突广泛融合，节段性内皮下及系膜区少量电子致密物沉积。给予舒洛地特软胶囊及氯沙坦钾片口服，奥雷巴替尼剂量未做调整，2022年11月复查尿蛋白（+），1年后尿蛋白转阴，目前奥雷巴替尼持续口服治疗中，融合基因持续测不出。

**图1:**
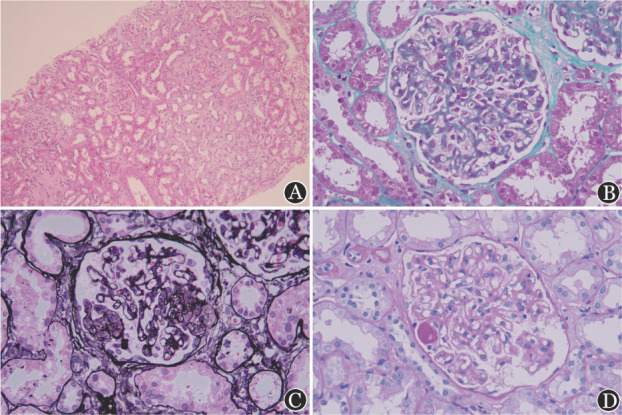
患者肾活检组织病理染色 **A** HE染色（×100）；**B** PAS染色（×400）；**C** PASM染色（×400）；**D** Masson染色（×400）

